# CYB561 promotes HER2+ breast cancer proliferation by inhibiting H2AFY degradation

**DOI:** 10.1038/s41420-024-01804-y

**Published:** 2024-01-20

**Authors:** Ting Zhao, Chaomin Wang, Na Zhao, Ge Qiao, Jialei Hua, Donghua Meng, Liming Liu, Benfu Zhong, Miao Liu, Yichao Wang, Changsen Bai, Yueguo Li

**Affiliations:** 1 Department of Clinical Laboratory, National Clinical Research Center for Cancer, Tianjin’s Clinical Research Center for Cancer, Key Laboratory of Breast Cancer Prevention and Therapy, Tianjin Medical University Ministry of Education, Key Laboratory of Cancer Prevention and Therapy, Tianjin, National Key Laboratory of Druggability Evaluation and Systematic Translational Medicine, Tianjin Medical University Cancer Institute and Hospital, Tianjin, China; 2 Department of Pathology, National Clinical Research Center for Cancer, Tianjin’s Clinical Research Center for Cancer, Key Laboratory of Breast Cancer Prevention and Therapy, Tianjin Medical University Ministry of Education, Key Laboratory of Cancer Prevention and Therapy, Tianjin, National Key Laboratory of Druggability Evaluation and Systematic Translational Medicine, Tianjin Medical University Cancer Institute and Hospital, Tianjin, China; 3 Department of Radiology, National Clinical Research Center for Cancer, Tianjin’s Clinical Research Center for Cancer, Key Laboratory of Breast Cancer Prevention and Therapy, Tianjin Medical University Ministry of Education, Key Laboratory of Cancer Prevention and Therapy, Tianjin, National Key Laboratory of Druggability Evaluation and Systematic Translational Medicine, Tianjin Medical University Cancer Institute and Hospital, Tianjin, China; 4 Department of Public Laboratory, National Clinical Research Center for Cancer, Tianjin’s Clinical Research Center for Cancer, Key Laboratory of Breast Cancer Prevention and Therapy, Tianjin Medical University Ministry of Education, Key Laboratory of Cancer Prevention and Therapy, Tianjin, National Key Laboratory of Druggability Evaluation and Systematic Translational Medicine, Tianjin Medical University Cancer Institute and Hospital, Tianjin, China; 5 Department of Pediatric Oncology, National Clinical Research Center for Cancer, Tianjin’s Clinical Research Center for Cancer, Key Laboratory of Breast Cancer Prevention and Therapy, Tianjin Medical University Ministry of Education, Key Laboratory of Cancer Prevention and Therapy, Tianjin, National Key Laboratory of Druggability Evaluation and Systematic Translational Medicine, Tianjin Medical University Cancer Institute and Hospital, Tianjin, China; 6 Department of Radiotherapy, National Clinical Research Center for Cancer, Tianjin’s Clinical Research Center for Cancer, Key Laboratory of Breast Cancer Prevention and Therapy, Tianjin Medical University Ministry of Education, Key Laboratory of Cancer Prevention and Therapy, Tianjin, National Key Laboratory of Druggability Evaluation and Systematic Translational Medicine, Tianjin Medical University Cancer Institute and Hospital, Tianjin, China; 7 Department of Clinical Laboratory Medicine, The First People’s Hospital of Xianyang, Xianyang, China

**Keywords:** Breast cancer, Cell growth

## Abstract

Breast cancer (BRCA) has a high incidence and mortality rate among women. Different molecular subtypes of breast cancer have different prognoses and require personalized therapies. It is imperative to find novel therapeutic targets for different molecular subtypes of BRCA. Here, we demonstrated for the first time that Cytochromeb561 (CYB561) is highly expressed in BRCA and correlates with poor prognosis, especially in HER2-positive BRCA. Overexpression of CYB561 could upregulate macroH2A (H2AFY) expression in HER2-positive BRCA cells through inhibition of H2AFY ubiquitination, and high expression of CYB561 in HER2-positive BRCA cells could promote the proliferation and migration of cells. Furthermore, we have demonstrated that CYB561 regulates H2AFY expression, thereby influencing the expression of NF-κB, a downstream molecule of H2AFY. These findings have been validated through in vivo experiments. In conclusion, we propose that CYB561 may represent a novel therapeutic target for the treatment of HER2-positive BRCA.

**Figure:**
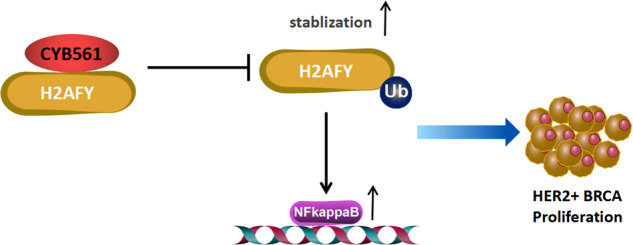
Graphical abstract CYB561 promotes the proliferation of HER2+ BRCA cells: CYB561 enhances the expression of H2AFY by inhibiting its ubiquitination, which leads to an increase expression of NF-κB in the nucleus. H2AFY, together with NF-κB, promotes the proliferation of HER2+ BRCA cells.

Graphical abstract CYB561 promotes the proliferation of HER2+ BRCA cells: CYB561 enhances the expression of H2AFY by inhibiting its ubiquitination, which leads to an increase expression of NF-κB in the nucleus. H2AFY, together with NF-κB, promotes the proliferation of HER2+ BRCA cells.

## Introduction

Breast cancer (BRCA) is the most common malignancy among women around the world [1], accounting for the fifth cause of cancer deaths among women in China [2]. The 13th St Gallen International Breast Cancer Conference (2013) defined three main categories and four subcategories of molecular typing, including luminal A/B, Erb-B2 overexpression (HER2-positive) and Basal-like (Triple negative) [3]. Different molecular subtypes of breast cancer, such as Luminal A, Luminal B, HER2-rich breast cancer, and triple-negative breast cancer (TNBC), exhibit diverse clinical behaviors, possess disparate prognoses, and necessitate tailored therapeutic approach [4]. Approximately 15–20% of human epidermal growth factor receptor 2 (HER2) is amplified and/or overexpressed in breast cancer [5]. HER2-positive BRCA is highly invasive, with short disease-free survival and poor prognosis. The HER2 gene (also known as HER2/neu, c-erbB-2) is located at 17q12 and is a member of the epidermal growth factor receptor (HER) family, which plays an important regulatory role in cellular physiological processes. The Luminal B BRCA subtype is characterized by its highly proliferative, metastatic, and often treatment-resistant nature, leading to a poor prognosis [6]. Triple-negative breast cancer has a worse prognosis than other subtypes, with a 10-year survival rate of less than 50% [7]. Therefore, it is imperative to find novel therapeutic targets for different molecular subtypes of BRCA.

Cytochrome b561(CYB561) belongs to cytochrome b561 family, member A1, located in 17q23. 3. Function of CYB561 is to transfer electrons, receive an electron from ascorbic acid and transfer this electron to the membrane [8]. Physiological functions facilitated by CYB561 encompass stress defense, cell wall modifications, iron metabolism, tumor suppression, and diverse neurological processes, such as memory retention [9, 10]. Studies reported that CYB561 is highly expressed in tissues of breast cancer patients and correlates with poor prognosis [11], and low expressed in endometrial cancer and ovarian cancer, also correlating with poor prognosis [12, 13]. However, these studies were bioinformatic analyses without experimental validation and mechanistic investigation.

H2AFY (also known as macroH2A1) is a histone H2A variant gene that plays roles in metabolic functions, transcriptional gene regulation, and DNA damage response [14]. H2AFY shows a high expression in many tumors, and it has been reported that overexpression of H2AFY induces cell proliferation through increased HER-2 activity [15]. In addition to that, H2AFY has a complex function in inflammatory responses and globally facilitating TNF-α-NF-κB signaling responses [16]. The transcription factor NF-κB consists of five subunits (p50, p52, RelA, RelB, c-Rel) and is regulated by the upstream activating kinase complex (IKKa, IKKb, and IKKc/NEMO). Notably, there exists an interactive relationship between HER2 and NF-κB, whereby HER2 can activate NF-κB through IKK, resulting in a wide-ranging pro-survival response, and NF-κB in turn can promote HER2 gene expression [17]. This positive feedback facilitates the progress of BRCA.

Here, we found that CYB561 is highly expressed in BRCA and correlates with poor prognosis, especially in HER2-positive BRCA. This high expression may contribute to the proliferation and invasion of BRCA cells. In addition, we found that CYB561 interacts with H2AFY. Interestingly, experimental demonstration that CYB561 can regulate H2AFY expression at the protein level and enhanced H2AFY protein stability, facilitated H2AFY nuclear expression, therefore fostering ectopic subcutaneous tumor proliferation. Our findings provide insight into the regulatory mechanisms underlying HER2-positive BRCA development and may provide a basis for the treatment of HER2-positive BRCA. Additionally, our results suggest that CYB561 could serve as a prognostic biomarker for HER2-positive BRCA.

## Results

### CYB561 is identified as a potential oncogene after data integration and implies poor prognosis of BRCA

**Fig. 1:**
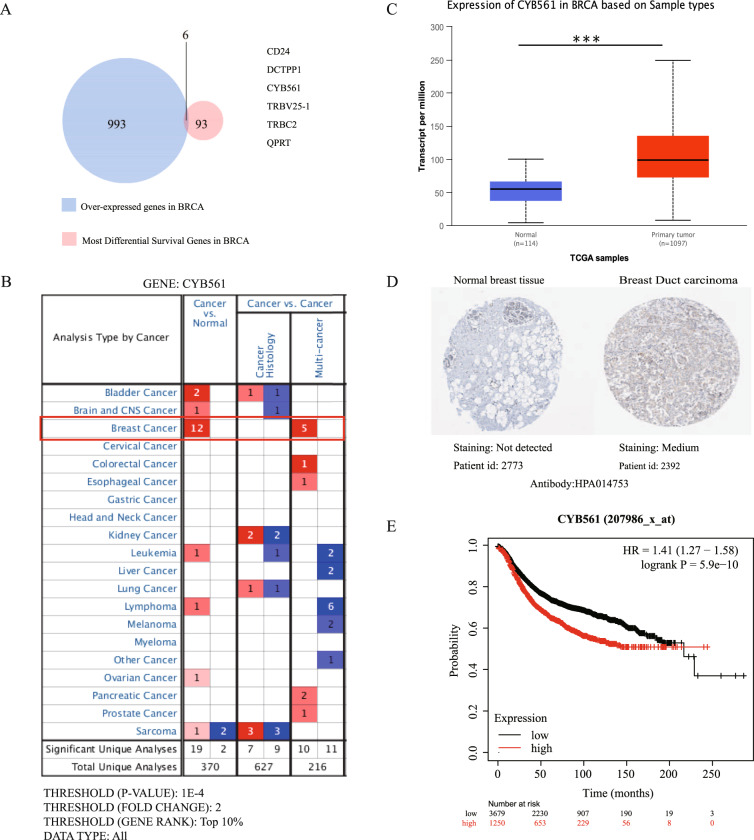
CYB561 is identified as a potential oncogene of BRCA after data integration. **A** Venn diagram of over-expression proteins and the most differentiated survival genes in BRCA (GEPIA database). **B** Analysis of CYB561 expression in BRCA (UALCAN database). **C** CYB561 mRNA level in 20 different types of primary tumor and normal tissues (ONCOMINE database). **D** CYB561 expression in BRCA tissues from HPA database. **E** The Kaplan–Meier survival analysis of overall survival (Kaplan-Meier Plotter database). ***p < 0.001. Statistical significance was determined by unpaired t-test.

To obtain the most important potential oncogene in BRCA, we analyzed the genes with the most differential mRNA expression and those most correlated with survival. From TCGA database, we obtained 1000 over-expressed genes and 100 most differential survival genes in BRCA. Finally, six overlapping genes (CD24, DCTPP1, CYB561, TRBV25-1, TRBC2, QPRT) from the above-mentioned two datasets were obtained (Fig. 1A). Candidate genes were selected after review of the published literature. In addition to CYB561, five other genes (CD24, DCTPP1, TRBV25-1, TRBC2, QPRT) have been previously reported multiple times in BRCA or other. Therefore, we aimed to interrogate the function of CYB561 in BRCA. CYB561 expression was analyzed in tumor and normal tissues of multiple cancers (total unique analyses *n* = 370). Among CYB561 over-expression cancer cases, the greatest numbers were BRCA (*n* = 12), the second was bladder cancer (*n* = 2). There was only one case of other cancers (Brain and CNS cancer, Leukemia, lymphoma, Ovarian, Sarcoma). In the comparison of cancer histology and multi-cancer of multiple cancers (*n* = 843, cancer histology =627, multi-cancer =216), BRCA was top ranking in multi-cancer (*n* = 5) (Fig. 1B). The mRNA expression of CYB561 in BRCA was significantly higher than normal through the UALCAN databases (Fig. 1C). Besides, expression of CYB561 in BRCA based on individual cancer stage, histology subtypes, patient’s race, menopause status were higher than the normal (Fig. S1A–D). The results of the Human Protein Atlas (HPA) database analysis showed that certain ductal and lobular carcinomas had elevated levels of CYB561 expression compared to normal tissues (Figs. 1D, S1E). However, the database lacks further molecular characterization. Moreover, Kaplan–Meier analysis indicated poorer prognosis in CYB561 high-expression samples (Fig. 1E). In conclusion, the elevated expression of CYB561 suggests its potential role as an oncogene, associated with a poorer prognosis.

### CYB561 has higher expression and shorter survival in HER2-positive BRCA patients

**Fig. 2:**
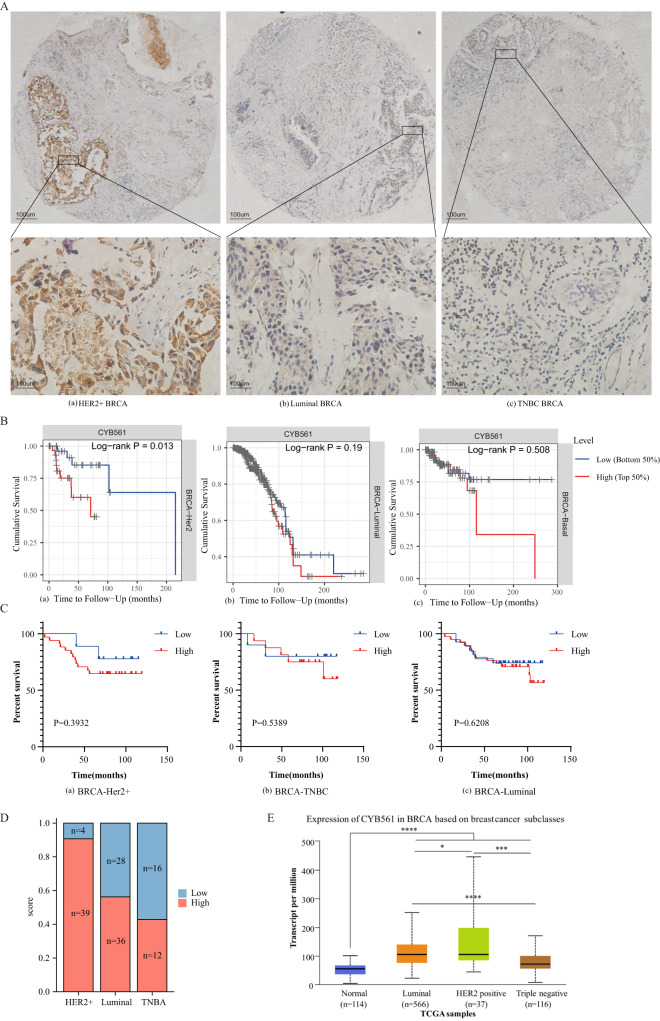
CYB561 has higher expression and shorter survival in HER2-positive BRCA patients. **A** IHC staining analysis of CYB561 protein in molecular subtyping of breast cancer tissues. The expression of CYB561 in HER2-positive BRCA, Luminal, and TNBC. **B** The survival of CYB561 expression level in different BRCA molecular subtyping (HER2-positive BRCA, Luminal, and TNBC) by data analysis. **C** The Kaplan–Meier survival analysis of CYB561 expression level in 140 BRCA tissue microarray specimens. **D** Percentage of different molecular typing in the CYB561 expression low and high subgroups. **E** Box plots of CYB561 expression in normal tissues and different BRCA molecular typing (HER2-positive BRCA, Luminal, and TNBC) from UALCAN database. *p < 0.05, ***p < 0.001, ****p < 0.0001. Statistical significance was determined by unpaired t-test.

BRCA were separated into four kinds of molecular subtypes (Luminal A, Luminal B, HER2-positive, and triple-negative BRCA) using immunohistochemical surrogates for molecular classifications [18]. To investigate the function of CYB561 in different molecular subtypes, the specimens were immunohistochemically stained. We found that the expression of CYB561 in HER2-positive BRCA was elevated compared to other molecular subtypes (Fig. 2A). High expression of CYB561 had poorer prognosis in HER2-positive BRCA than other molecular subtypes from TIMER database (Fig. 2B). Besides, patients with HER2-positive BRCA who exhibited high expression of CYB561 in tissue microarray data demonstrated an unfavorable prognosis; however, the lack of statistical significance in this observation may be attributed to the limited sample size (Fig. 2C). The rate of CYB561 positivity was shown in a histogram. Tissue microarray data showed a higher percentage of CYB561 expression in HER2-positive BRCA (Fig. 2D). Transcript analysis was implemented on human datasets publicly available through the TCGA database. Expression of CYB561 showed the highest levels in HER2-positive BRCA (Fig. 2E). Consequently, our results revealed that high expression of CYB561 clearly correlated with poor prognosis in HER2-positive BRCA.

### CYB561 over-expression contributes to cell proliferation of HER2-positive BRCA

**Fig. 3:**
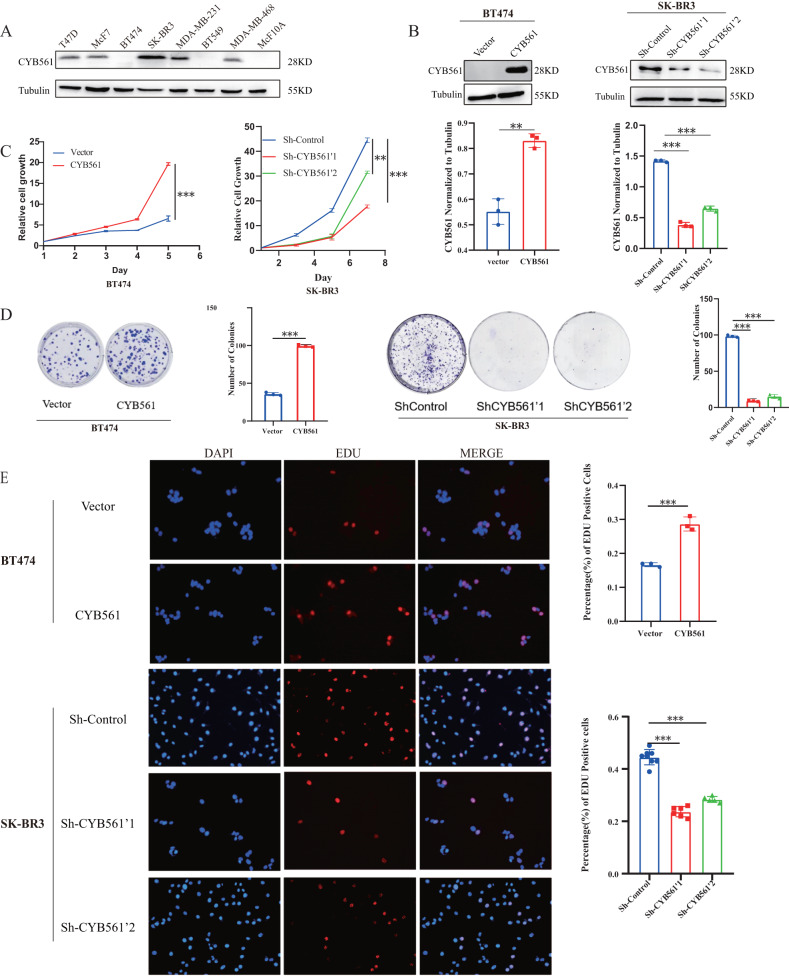
CYB561 over-expression contributes to cell proliferation of HER2-positive BRCA. **A** Expression of CYB561 in commonly used breast cancer cell lines was detected by western blot. **B** Western blotting was performed to determine levels of CYB561 protein. Expression of CYB561 relative to Tubulin was quantified. **C** Detection of proliferative capacity of breast cancer cells after overexpression or knockdown of CYB561. **D** Colony formation assay showing the effects of CYB561 overexpression or knockdown on BRCA cells. **E** EDU assay for proliferation of cells overexpressing or knocking down CYB561-treated cells. **p < 0.01, ***p < 0.001. Statistical significance was determined by unpaired t-test.

CYB561 was more likely to boost HER2-positive BRCA cell proliferation based on the results above. Subsequent experiments focused on HER2-positive BRCA. Firstly, detection of CYB561 expression was performed in common breast cancer cell lines and 293T cells (Fig. 3A). Notably, normal mammary epithelial cells MCF10A exhibited minimal expression of CYB561. As a commonly used tool cell, CYB561 was highly expressed in 293T cells (Fig. S2A). The HER2-positive BRCA cell lines BT474 and SKBR-3, which displayed low and high expression of CYB561, respectively, were selected for further experimentation involving overexpression and knockdown techniques (Fig. 3B). The proliferation experiments showed that over-expression of CYB561 facilitated cell proliferation, while knockdown of CYB561 suppressed cell viability (Fig. 3C). Moreover, cell proliferation was assayed by colony formation. Results indicated that over-expression of CYB561 strengthen colony formation, while knockdown of CYB561 inhibited colony formation (Fig. 3D). To detect proliferating cells, we also used the Edu assay, which stains S-phase cells, which led to similar conclusions (Fig. 3E). Interestingly, we also found that CYB561 promoted BRCA cell migration (Fig. S3A, B). In summary, high expression of CYB561 facilitated cell proliferation of HER2-positive BRCA.

### CYB561 interacts with H2AFY

**Fig. 4:**
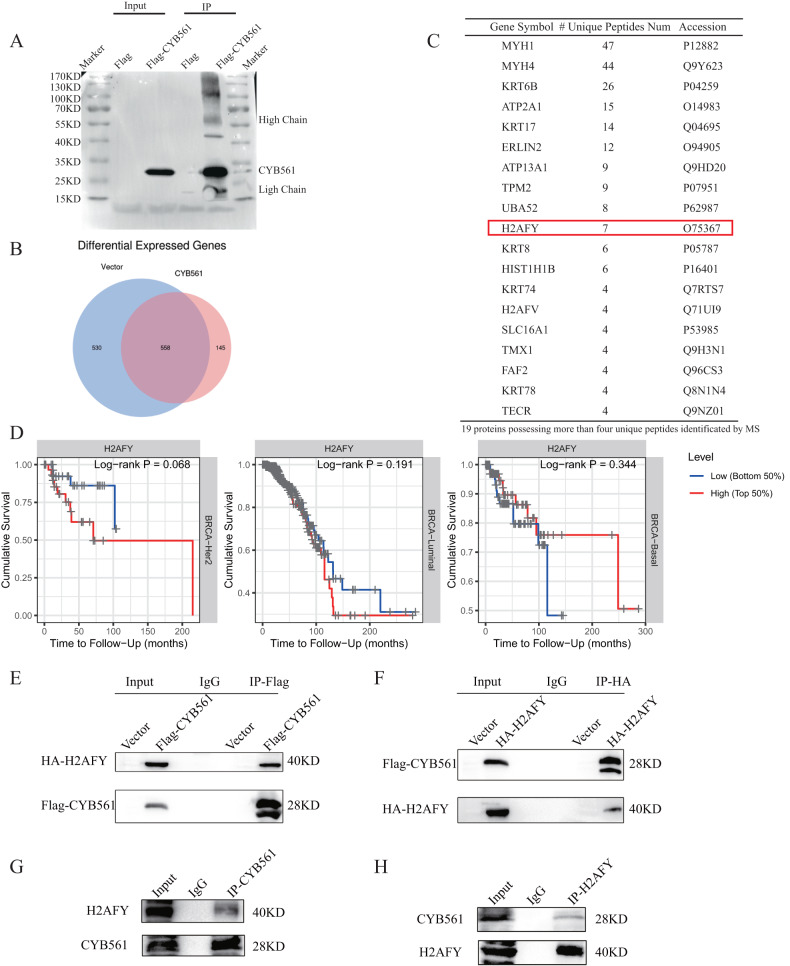
CYB561 interacts with H2AFY. **A** Immunoprecipitation (IP) in 293 T cells with CYB561-overexpression. **B** Venn diagram showing the differential proteins detected by mass spectrometry. **C** A total of 19 differential proteins with more than identified four unique peptides by mass spectrometry. **D** The survival of H2AFY expression level in different BRCA molecular subtyping (HER2-positive BRCA, Luminal, and TNBC) by data analysis (TIMER 2.0 database). **E**, **F** Validation of CYB561 interaction with H2AFY by exogenous IP. **G**, **H** Validation of CYB561 interaction with H2AFY by endogenous IP.

In further dissecting the cancer-promoting mechanisms of CYB561 and identifying the potential substrates of CYB561, we overexpressed CYB561 in HEK293T cells, HEK 293T cells that were transfected with an empty vector were shown as a control. To identify CYB561-interacting proteins, we performed semi-quantitative mass spectrometry proteomic analysis of CYB561 immunoprecipitations (IPs) products (Fig. 4A). A large number of potential CYB561-interacting proteins were identified. A total of 145 proteins were found to likely interact with CYB561 compared to control tissue after analyzing mass spectrometry results (Fig. 4B). All proteins identified with less than four unique peptides were excluded from analysis. A total of 19 molecule candidates were selected (Fig. 4C). Prognostic analysis was performed on each molecule candidate. Analysis results indicated that H2AFY high-expression possessed shorter survival in HER2 + BRCA (p = 0.068), consistent with the prognosis of CYB561. There was no significant difference in survival in other BRCA molecular typing. There was virtually no difference in all other molecule candidates among BRCA molecular typing (Fig. 4D, S4). Previous studies have reported that H2AFY interacted with HER2 and derived tumorigenicity [15].Based on these considerations, we leave H2AFY as a preferred for further research. Exogenous and endogenous co-IP experiments demonstrated that CYB561 interacted with H2AFY (Fig. 4E–H). In summary, H2AFY was one of the interacting molecules of CYB561 after mass spectrometry analysis and experimental verification.

### CYB561 regulates expression of H2AFY by inhibiting its ubiquitination

**Fig. 5:**
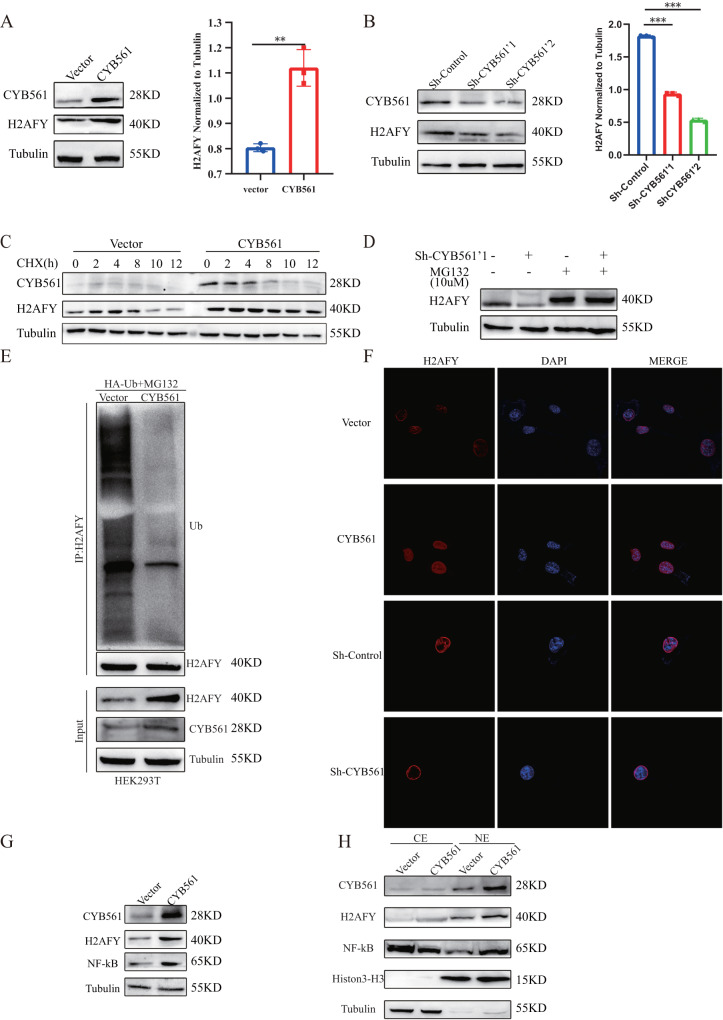
CYB561 inhibits the ubiquitination of H2AFY. **A**, **B** Immunoblotting of H2AFY expression in CYB561 overexpressed or knocked down in BRCA cells. **C** CYB561 overexpressed cells were treated with CHX and collected at the indicated time. **D** CYB561-knocked down cells were treated with or without MG132. Immunoblotting analysis of H2AFY expression. **E** CYB561 overexpressed cells were transfected with HA-Ub and treated with MG132 for 12 h. Extracts were immunoprecipitated with anti-H2AFY agarose beads, followed analysis by immunoblotting. **F** Immunofluorescence of H2AFY expression in CYB561 overexpressed or knocked down in BRCA cells. **G** Immunoblotting of NF-κB and H2AFY expression in CYB561 overexpressed cell. **H** Cell fractionation assays revealed that overexpression of CYB561 increased the nuclear H2AFY and NF-κB protein levels. **p < 0.01, ***p < 0.001. Statistical significance was determined by unpaired t-test.

In order to ascertain the regulatory connections between CYB561 and H2AFY, we conducted a validation of the protein and RNA levels of both CYB561 and H2AFY. The findings revealed that CYB561 was overexpressed, resulting in an elevation of H2AFY levels (Fig. 5A). Conversely, the knockdown of CYB561 resulted in a decrease in H2AFY protein levels in SKBR3 cells (Fig. 5B). Notably, the mRNA levels of H2AFY remained unchanged following both CYB561 overexpression and knockdown (Fig. S5A, B). Consequently, it can be concluded that CYB561 regulates the expression of H2AFY at the protein level rather than the mRNA level. Therefore, we speculated that CYB561 regulated H2AFY by one of post-translational modifications. We found H2AFY was modified by ubiquitination after extensive review of literatures [19]. Ubiquitination, the covalent attachment of ubiquitin to target proteins, is one of the most versatile post-translational modifications in the cell as it can modify substrate proteins in its monomeric form (monoubiquitylation) or be conjugated in the form of ubiquitin chains (polyubiquitination) [20]. We performed GO molecular function enrichment analysis on CYB561 interacting partners. The enrichment result indicated that enriched molecular functions including ubiquitination (Fig. S5C). Meanwhile, protein synthesis inhibitor cycloheximide (CHX) was used to impede protein synthesis and the protein half-life of H2AFY was determined. Overexpression of CYB561 extended protein half-life of H2AFY (Fig. 5C). In addition, proteasomal inhibitor MG132 could restore H2AFY expression (Fig. 5D), which suggested that H2AFY was stabilized by CYB561 through the ubiquitin (Ub) proteasome system (UPS). These findings suggest that CYB561 may be involved in the deubiquitylation of H2AFY. Consistently, CYB561 overexpression decreased H2AFY ubiquitination in HEK293T cells (Fig. 5E). Furthermore, obviously stronger nuclear signals of H2AFY were detected when the expression of exogenous CYB561 was induced, while the nuclear signals of H2AFY were significantly decreased in CYB561-knockdown cells (Fig. 5F). Besides, previous literatures have reported H2AFY facilitated the response of cancer cells to TNFα-NF-κB pathway [16]. GO biological Process enrichment demonstrated the genes interaction with CYB561 enriched on NF-κB signaling (Fig. S5D). Compared with the control cells, the level of NF-κB was increased in CYB561-overexpressing cell (Fig. 5G). Using cell fractionation and Western blot, we found that both the cytoplasmic level of NF-κB was decreased, the nuclear level of NF-κB was significantly increased upon CYB561 overexpression (Fig. 5H). Therefore, these results indicated that CYB561 regulated expression of H2AFY by inhibiting its ubiquitination.

### CYB561 plays an important role in cancer‐promoting by regulating H2AFY

**Fig. 6:**
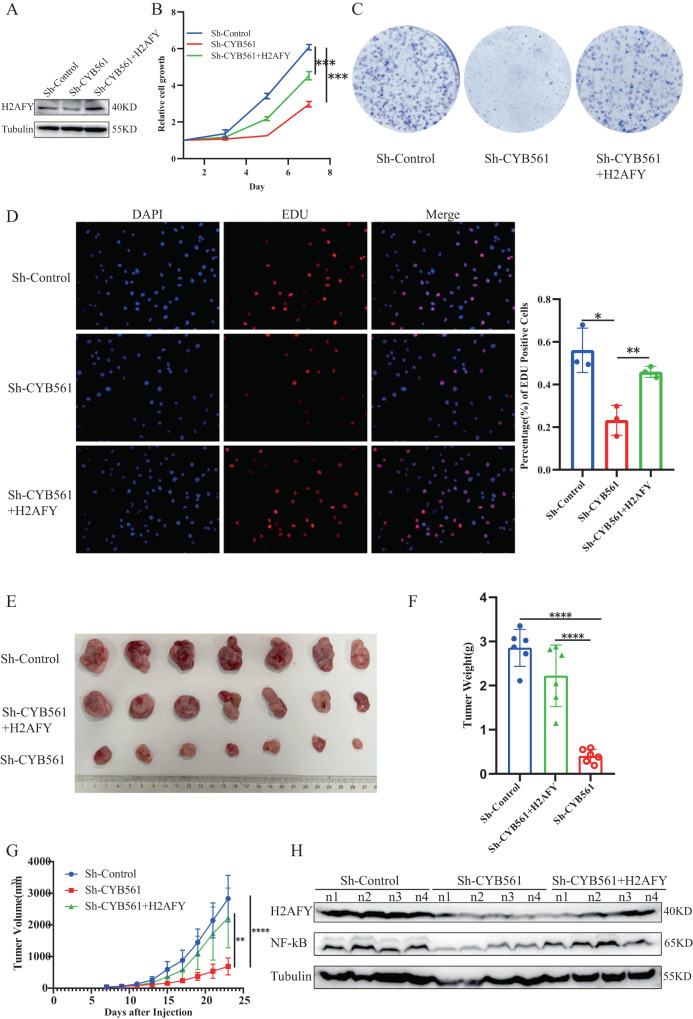
Knocking down CYB561 while replenishing H2AFY rescues cell proliferation in vivo and in vitro. **A** Western blotting was performed to determine levels of H2AFY protein. **B** Cell proliferation was detected in CYB561-knocked down cell transfected with H2AFY. **C** Colony forming efficiency of CYB561-knocked down cell transfected with H2AFY. **D** EDU assay was used to detect cell proliferation capacity of CYB561-knocked down cell transfected with H2AFY. **E** Images of tumors from nude mice in Sh-Control, ShCYB561 and ShCYB561 adding back of H2AFY groups. **F**, **G** Tumor weights and volumes in the three groups. **H** The protein level of H2AFY and NF-κB in the established xenograft tissue model was assessed by Western Blotting. *p < 0.05, **p < 0.01, ***p < 0.001, ****p < 0.0001. Statistical significance was determined by unpaired t-test.

Previous literatures have reported H2AFY enhanced cell proliferation [21]. We used the knockdown and rescue approach to validate whether CYB561 promoted proliferation by regulating expression of H2AFY. Firstly, we overexpressed H2AFY on the basis of knockdown of CYB561 stably transduced cell lines (Fig. 6A). We found that knocking down of CYB561 reduced growth of the cells, consistent with our previous findings, over-expression of H2AFY rescued growth of the CYB561 knockdown cells (Fig. 6B). Besides, replying to the expression of H2AFY rescued the clonogenic ability of breast cancer cells (Fig. 6C). Equally, the EDU result showed that the abundance of H2AFY restored cell proliferation in SK-BR3 (Fig. 6D). To investigate the role of CYB561 in tumor growth in vivo, SK-BR3 cells with CYB561 knockdown, along with negative control cells, were subcutaneously injected into nude mice for subsequent growth analysis. The findings revealed that the growth of the subcutaneous human BRCA xenograft in the CYB561 knockdown group was significantly suppressed (Fig. 6E). Additionally, both tumor weight and volume exhibited a notable decrease (Fig. 6F, G). The tumor growth, volume and weight in vivo were rescued after over-expression H2AFY (Fig. 6E–G). To filtrate CYB561 promoting cancer mechanisms, we investigated the protein level of H2AFY and NF-κB in subcutaneous tumors. Knockdown of CYB561 expression led to a subsequent reduction in H2AFY and NF-κB levels. Conversely, overexpression of H2AFY resulted in the restoration of NF-κB expression (Fig. 6H). In summary, our findings suggest that CYB561 facilitates the proliferation of HER2 + BRCA cells by enhancing the abundance of H2AFY and promoting the accumulation of NF-κB.

## Discussion

BRCA has emerged as the predominant form of cancer among women, surpassing lung cancer in terms of incidence rates [22]. Notably, patients with ERBB2-amplified or overexpressing breast cancer can derive therapeutic benefits from ERBB2-targeted interventions, encompassing anti-ERBB2 antibodies (e.g., trastuzumab and epratuzumab) and small-molecule tyrosine kinase inhibitors (e.g., lapatinib and neratinib) [23]. However, it is important to acknowledge that young women afflicted with breast cancer face heightened vulnerability to sexual and psychological distress [24]. Therefore, investigating the mechanisms of breast cancer development and identifying potential therapeutic targets are the focus of research. Over the past 20 years, breast cancer screening in China has begun to gain traction and has gradually become standardized. Molecular typing of breast cancer not only precisely assigns breast cancer patients to different prognostic groups, but also provides guidance for treatment [25, 26]. In this study, we found that CYB561 was overexpressed in HER2-positive BRCA patients and was correlated with poor survival. CYB561 significantly promoted HER2-positive BRCA proliferation in vitro and in vivo, which regulates the expression of H2AFY by inhibiting its ubiquitination. These results suggest that CYB561 could be a potential target for the therapy of HER2-positive breast cancer.

In past decades, several research studies have shown that the critical role of CYB561 plays in sympathetic function and cardiovascular regulation [27]. In contrast, there are few studies on the function of CYB561 in the oncology field. Studies have identified CYB561 as a therapeutic target and biomarker in neuroendocrine prostate cancer cells, and highlight the potential use of that can be used to identify more aggressive disease [28]. In 2022, bioinformatic analysis has reported that CYB561 expression and clinical prognostic value in breast cancer [11]. However, the mechanism of action of CYB561 in tumors has been poorly studied. In the present study, our data analysis demonstrated that CYB561 expression was higher in breast cancer than in non-tumor tissues, high expression of CYB561 is associated with a shorter survival period in patients with BRCA. Furthermore, the immunohistochemical staining results and subsequent data analysis revealed that CYB561 exhibited a higher expression level and was associated with a poorer prognosis specifically in HER2-positive breast cancer (BRCA) compared to other molecular subtypes of BRCA.

Evidence from recent decades has indicated that H2AFY proteins play an important role in many aspects of cancer development and progression. While some studies have provided evidence that H2AFY inhibits tumor progression, other studies have highlighted potential pro-tumor functions [29]. Previously reported that H2AFY was highly expressed in hepatocellular carcinoma, triple negative breast cancer, colon cancer, lymphoma and melanoma and promoted tumor proliferation [30–34]. In HER2-positive BRCA, HER2 induced expression of H2AFY, interacted with H2AFY, amplified HER2 activity, suggesting a potential positive feedback loop. Our study indicated that the upregulation of CYB561 leads to an increase in the expression of H2AFY. Conversely, H2AFY decreased after knockdown of CYB561. Therefore, we argued that CYB561 performs a pro-cancer function in HER2-positive breast cancer by regulating the expression of H2AFY at protein level. Interestingly, we found CYB561 maintained H2AFY stability by inhibiting H2AFY ubiquitination degradation. It is worth noting that previous studies have also reported the involvement of ubiquitination in the regulation of H2AFY [35]. Further studies will be aimed at investigating the molecular mechanism by which CYB561 inhibits the ubiquitination of H2AFY and, thereby, suppressed its degradation. NF-κB is extensively involved in the regulation of BC proliferation, drug resistance, angiogenesis and metastasis [36]. Overexpression of NF-κB ultimately leads to enhanced expression of NF-κB-responsive genes, which contribute to BC progression. For example, cell cycle protein E, one of the target genes of NF-κB, is highly expressed in many BC cell lines and is associated with poor prognosis [37]. We also found that CYB561 regulated the expression of NF-κB. It is interesting to note that H2AFY facilitated the response of cancer cells to TNFα-NF-κB [16]. We argued that CYB561 regulated the protein expression of H2AFY and thus promoted the expression of the NF-ΚB axis.

In summary, our study has demonstrated that CYB561, functioning as an oncogene, exerts a significant influence on the rapid proliferation and growth of HER2-positive BRCA cells, both in vitro and in vivo. Furthermore, we have identified that CYB561 plays a crucial role in modulating H2AFY and maintaining its stability through ubiquitination regulation. This, in turn, leads to the upregulation of the downstream molecule NF-κB, thereby contributing to the accelerated development of HER2-positive breast cancer. Consequently, the CYB561-H2AFY-NF-κB signaling pathway may hold substantial importance in impeding the progression of HER2-positive BRCA.

## Materials and methods

### Cell culture and tissues

HEK293T, SK-BR3, and BT474 cells were obtained from American Type Culture Collection (ATCC, Manassas, VI) and cultivated in DMEM or 1640 medium (Corning, NY, USA), supplemented with 10% fetal bovine serum (FBS; PAN-Seratech, Edenbach, Germany) and 1% penicillin/streptomycin (PS; HyClone, Logan, UT, USA) in a humidified atmosphere with 5% CO_2_ at 37 °C. All cell lines were cultured for no more than 2 months and were confirmed to be free of mycoplasma contamination. Tissue chips were purchased from Shanghai Outdo Biotechnology Co.

### Plasmids and transfection

The CYB561 overexpression plasmid and lentivirus were purchased from GeneChem, Shanghai. CYB561 shRNA was used to knockdown this gene. The shRNA sequences used in this study were shCYB561 #1 GAGTCCCTCCAGCCTGAATAA shCYB561#2 GGGCAAGTATAGCGCATTTGA. Viral packaging was performed according to a previously described protocol [38]. HEK293T cells were co-transfected with packaging plasmids (pCMV-dR8.91 and pCMV-VSV-G) and expression plasmids (Sh-Control, Sh-CYB561, vector and H2AFY) using PEI (Polysciences, cat. 23966-2) to produce lentivirus. Cells were infected with the viruses at a titer of 100% infection in the presence of polybrene (10 μg/ml) for 48 h, after which positive cells were selected using puromycin for 7 days, 5 mg/mL (SK-BR3) and 1 mg/mL (BT474). Western blotting validated transfection efficiency.

### Immunohistochemistry

The human BRCA tissue microarrays were baked in a 65 °C oven for 2 h and rehydrated with dewaxing in gradient decreasing concentrations of xylene and ethanol in sequence. The antigen was extracted with citrate buffer (ZSGB-BIO, ZLI-9065) for 90 seconds in a pressure cooker in order to expose the epitope to the antibody.Inhibit endogenous peroxidase activity with 3% hydrogen peroxide. The membrane was incubated with goat serum at 37 °C for half an hour. Subsequently, tissue microarrays were incubated with rabbit anti-CYB561(1:50, ThermoFisher, PA5-53228) overnight at 4 °C. Tissue microarrays were washed with phosphate buffered saline (PBS) and stained with secondary antibody at 37 °C for 1 h. Then microarrays were stained with diaminobenzidine solution (Zhongshan Biotechnology, Beijing, China) following by re-staining with hematoxylin. Tissue microarrays were photographed using an optical microscope (Olympus BX61). The final IHC score for CYB561 was the sum of the product of the staining intensity score and the percentage score. HER2 status was determined by FISH results. HER2 status was positive for HER2 + BRCA. Estrogen receptor (ER) and progesterone receptors (PR) nuclear ≥5% was positive. ER, PR, HER2 status were negative for TNBC. The others belonged to Luminal BRCA.

### Western blot and antibodies

The cells were lysed on ice for 30 min after discarding the medium with 1× SDS lysis buffer replenishing with protease inhibitor cocktail (Roche, 11873580001), 1 mM NaF, 1 mM Na_3_VO_4_. Collected the proteins and boiled them in a metal bath at 95 °C for 15 min. Gel electrophoresis with equal amount of protein under constant voltage of 70 V or 130 V on PAGE gel. Subsequently, the proteins were transferred to polyvinylidene fluoride membrane (PVDF, Immobilon-P, Millipore, Billerica, MA, USA) and blocked with 5% bovine serum albumin to block non-specifics binding for 1 h at room temperature. Then, incubated PVDF membrane with primary antibody at 4 °C overnight and secondary antibody for 1 h at room temperature, followed by chemiluminescence detection by ECL (Pierce, Rockford, IL, USA). The antibodies used include the following: anti-CYB561(Cat #PA5-53228,1:1000, Thermo Fisher), anti-Tubulin (Cat #2146, 1:1000, Cell Signaling Technology, anti-H2AFY (Cat #26875-1-AP,1:1000, Proteintech), anti-NF-κB (Cat #sc-8008,1:1000, Santa Cruz Biotechnology), HRP-conjugated anti-rabbit (Cat #sc-2357,1:4000, from Santa Cruz Biotechnology), Ubiquitin (Cat ##3936,1:1000, Cell Signaling Technology).

### Immunofluorescence

A total of 2 × 10^4^ cells/well were seeded onto immunofluorescent slides in twelve-well plates. The cells recovered their morphological form after 24 h, were fixed with 4% paraformaldehyde for 15 min, and then were washed with PBS and were permeabilized with 0.2% Triton X-100 in PBS for 15 min at room temperature. Next, immunofluorescent slides with cells were incubated with primary antibody overnight at 4 °C, followed by secondary antibodies for 1 h at room temperature. The nuclei were stained with DAPI (1:1000) subsequently. Finally, the films were sealed with anti-quenching sealer. Confocal imaging was performed with a ZEISS LSM880 Confocal Microscope at 63 magnifications.

### Quantitative real-time PCR (qRT-PCR)

Total RNA was extracted by TRIzol reagent (Ambion, USA). Nest, reversed transcription of RNA to cDNA using the PrimeScript™ RT MasterMix kit (Takara, Japan). qRT-PCR was performed using AceQ SYBR qPCR Master Mix (Vazyme, China) according to the manufacturer’s instructions. The primers sequences are used as follows: CYB561 forward primer: GTCTTCAGGAACGAAGCTAAAC, reverse primer: CTTCTTCCTGTGGTAGTCGAAC. H2AFY forward primer: TGCTGCGGTACAT CAAGAAAGGC, reverse primer: CCCCACTGGCTATGGTGACTCC.

### Proliferation and colony formation assays

Cell proliferation assay was performed according to the manufacturer’s instructions using the BeyoClick™ EdU-594 Cell Proliferation Kit (Beyotime Biotech. Inc.) Breifly, a total of 1 × 10^4^ cells/well were seeded in 12-well plates. Cells were digested with trypsin on the third, fifth, seventh day in succession. Live cells were counted by light microscopy using a Neubauer hemocytometer. The Counting was performed in triplicate and repeated at least three times. A total of 1000 cells/well were seeded in 6-well plates for the colony formation assay. Cells were cultured for two weeks at 37 °C incubators, fixed in 4% paraformaldehyde (Solarbio, Beijing, China) and stained with 1% crystal violet (Sigma-Aldrich).

### IP and mass spectrometry

Cells were harvested at an 80% fusion rate and rinsed three times with pre-cold PBS. Then, cells were lysed in IP lysis buffer (0.3%IP buffer:10%NP-40, 2. 5 M NaCl, 1MTris-HCl PH7. 5, 0. 5M EDTA, DDW) on ice for 1 h. The lysate was centrifuged at 12,000 rpm and removed the supernatant in new tubes. The beads (ANTI-FLAG® M1 Agarose Affinity Gel, Sigma-Aldrich) washed with lysate were mixed with cell lysate, overnight at 4 °C. Lysates of IP were further verified and analyzed by western blotting and MS.

### Animal models

Female BALB/c nude mice (4 weeks old) were purchased from GemPharmatech. Cells were tryptic digested and counted 5 × 10^6^ before subcutaneous injection. Nude mice, after subcutaneous injection were observed every other day. The tumor sizes were measured with vernier calipers at 7 days after injection. The mice were euthanized after 6 weeks, at which time the subcutaneous tumors were dissected. Tumors were weighed and standard histologic examination was performed. The Animal Care and Use Committee of Tianjin Medical University evaluated and approved the experimental protocol.

### Bioinformatics data analysis

The Kaplan–Meier plotter database (http://kmplot.com/analysis/) and TIMER2. 0 online tool (https://cistrome.shinyapps.io/timer/) were used to survival analyses. The differential expression analysis of CYB561 was determined using Oncomine (https://www.oncomine.com/), GEPIA (http://gepia.cancer-pku.cn/) and UALCAN (http://ualcan.path.uab.edu/) database. The immunohistochemical maps of CYB561 were determined using HPA (https://www.proteinatlas.org/). The mRNA expression and survival of genes in BRCA from the TCGA database were analyzed using xiantao tools (https://www.xiantaozi.com). The analytical approach employed for real-time fluorescence quantitative data analysis was the 2^−ΔΔCt^ method (Livak method), which is a relative quantitative method. Proportional risk hypothesis testing was conducted using the survival package and fitted survival regressions, with the outcomes presented through the utilization of the survminer package in conjunction with the ggplot2 package.

### Statistical analysis

Two-tailed Student’s *t* test was used to analysis differential comparisons among the groups. Statistical analyses were performed using SPSS 25. 0 or GraphPad Prism 8.0.1. Independent risk factors and survival curves were analyzed using the one-factor Kaplan-Meier method and the multi-factor Cox method. ImageJ was used for analysis and quantifications. Statistical significance was defined as *p* <0.05.

### Supplementary information

Supplemental figure legend

supplementary Figure 1

supplementary Figure 2

supplementary Figure 3

supplementary Figure 4

supplementary Figure 5

Original Data File

## Data Availability

Datasets used and/or analyzed during the current study are available from the corresponding author upon reasonable request.
