# Supplementary material for: CYB561 promotes HER2+ breast cancer proliferation by inhibiting H2AFY degradation
Source: Cell Death Discov. 2024 Jan 20;10:38. doi: 10.1038/s41420-024-01804-y (PMC10799939; doi:10.1038/s41420-024-01804-y)
Supplement: Supplementary file 1 — Supplemental figure legend [file 41420_2024_1804_MOESM1_ESM.docx]

**Figure S1 CYB561 is identified as a potential oncogene of BRCA**

1. D) Expression of CYB561 in BRCA based on individual cancer stage, histology subtypes, patient's race, menopause status. The Kaplan–Meier survival analysis of DFBS, RFS (Kaplan-Meier Plotter database). (E) CYB561 expression in BRCA tissues from HPA database. *p < 0. 05, **p < 0. 01, ***p < 0. 001, ****p < 0. 0001.

**Figure S2 CYB561 has higher expression in HER2-positive BRCA cells.**

(A)Protein expression levels of CYB561 in 293T and HER2-positive BRCA cells.

**Figure S3 CYB561 promotes the migration of HER2-positive cells.**

(A, B) Scratch assay showing the effect of CYB561 overexpression or knockdown on the migratory capacity of BRCA cells. *p < 0. 05, **p < 0. 01, ***p < 0. 001.

**Figure S4 Bioinformatics data analysis of candidate molecules.**

Survival of candidate molecule expression levels in different BRCA molecular subtypes (HER2-positive BRCA, Luminal and TNBC) by data analysis (TIMER 2.0 database).

**Figure S5** **CYB561 inhibits the ubiquitination of H2AFY.** (A)The mRNA expression levels of CYB561 and H2AFY after overexpression of CYB561. (B)The mRNA expression levels of CYB561 and H2AFY after knockdown of CYB561. (C) Go functional enrichment analysis of protein molecules interacting with CYB561. (D) Go biological process enrichment analysis of proteins interacting with CYB561.
