# Supplementary material for: CYB561 promotes HER2+ breast cancer proliferation by inhibiting H2AFY degradation
Source: Cell Death Discov. 2024 Jan 20;10:38. doi: 10.1038/s41420-024-01804-y (PMC10799939; doi:10.1038/s41420-024-01804-y)
Supplement: Supplementary file 2 — supplementary Figure 1 [file 41420_2024_1804_MOESM2_ESM.pdf]

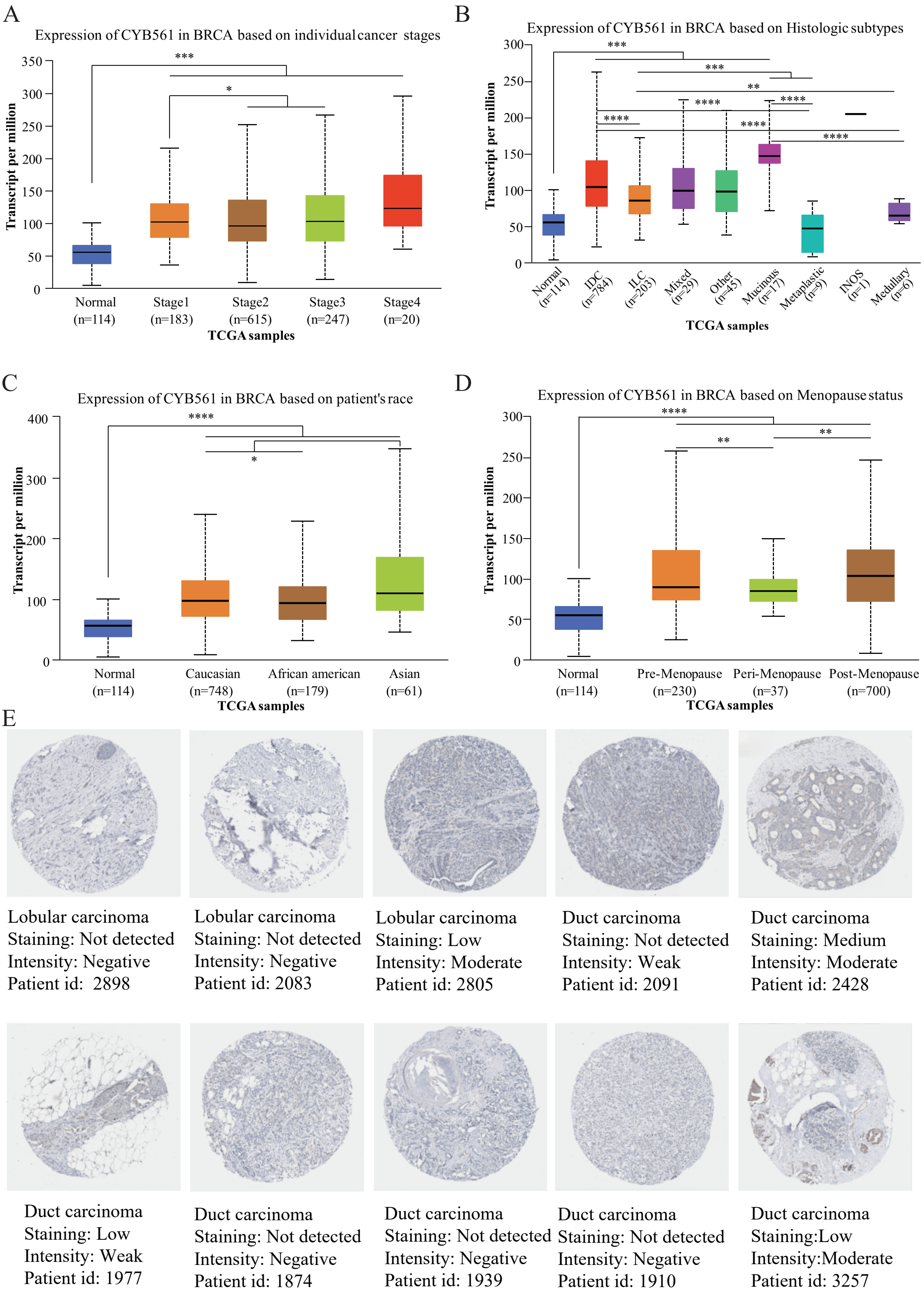

A
Expression of CYB561 in BRCA based on individual cancer stages
Transcript per million
Normal (n=114)
Stage1 (n=183)
Stage2 (n=615)
Stage3 (n=247)
Stage4 (n=20)
TCGA samples
B
Expression of CYB561 in BRCA based on Histologic subtypes
Transcript per million
Normal (n=114)
IDC (n=784)
ILC (n=203)
Mixed (n=29)
Other (n=45)
Mucinous (n=17)
Metaplastic (n=9)
INOS (n=1)
Medullary (n=6)
TCGA samples
C
Expression of CYB561 in BRCA based on patient's race
Transcript per million
Normal (n=114)
Caucasian (n=748)
African american (n=179)
Asian (n=61)
TCGA samples
D
Expression of CYB561 in BRCA based on Menopause status
Transcript per million
Normal (n=114)
Pre-Menopause (n=230)
Peri-Menopause (n=37)
Post-Menopause (n=700)
TCGA samples
E
Lobular carcinoma
Staining: Not detected
Intensity: Negative
Patient id: 2898
Lobular carcinoma
Staining: Not detected
Intensity: Negative
Patient id: 2083
Lobular carcinoma
Staining: Low
Intensity: Moderate
Patient id: 2805
Duct carcinoma
Staining: Not detected
Intensity: Weak
Patient id: 2091
Duct carcinoma
Staining: Medium
Intensity: Moderate
Patient id: 2428
Duct carcinoma
Staining: Low
Intensity: Weak
Patient id: 1977
Duct carcinoma
Staining: Not detected
Intensity: Negative
Patient id: 1874
Duct carcinoma
Staining: Not detected
Intensity: Negative
Patient id: 1939
Duct carcinoma
Staining: Not detected
Intensity: Negative
Patient id: 1910
Duct carcinoma
Staining:Low
Intensity:Moderate
Patient id: 3257
