# Supplementary figures and images for: CYB561 promotes HER2+ breast cancer proliferation by inhibiting H2AFY degradation
Source: Cell Death Discov. 2024 Jan 20;10:38. doi: 10.1038/s41420-024-01804-y (PMC10799939; doi:10.1038/s41420-024-01804-y)

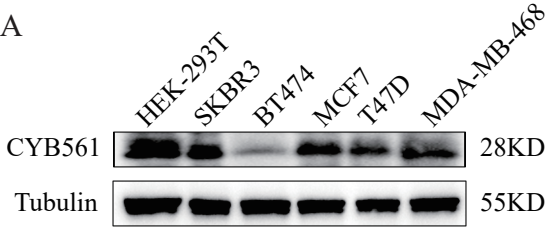

Supplement: Supplementary file 3 — supplementary Figure 2 [file 41420_2024_1804_MOESM3_ESM.pdf]

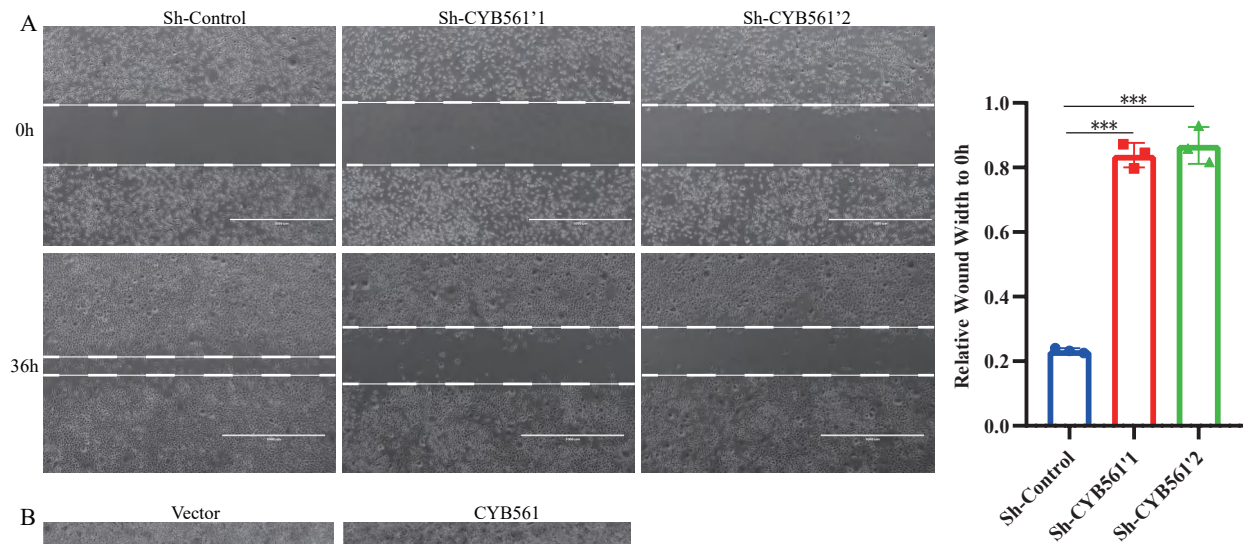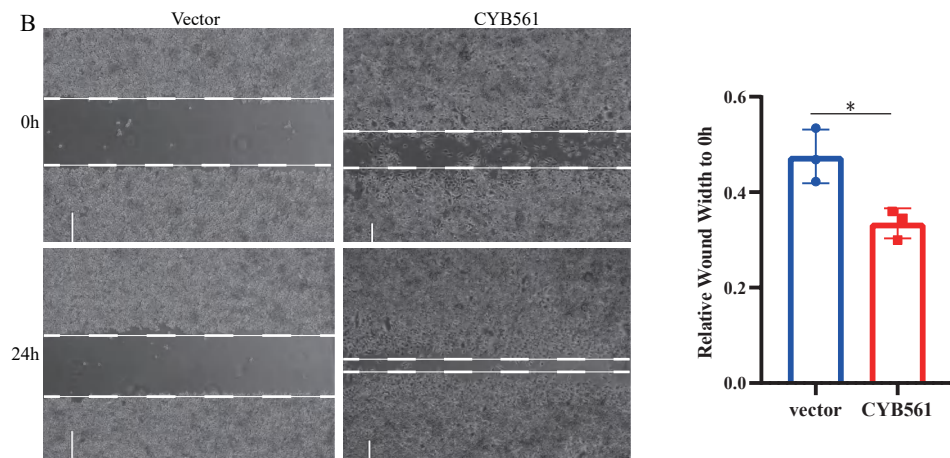

Supplement: Supplementary file 4 — supplementary Figure 3 [file 41420_2024_1804_MOESM4_ESM.pdf]

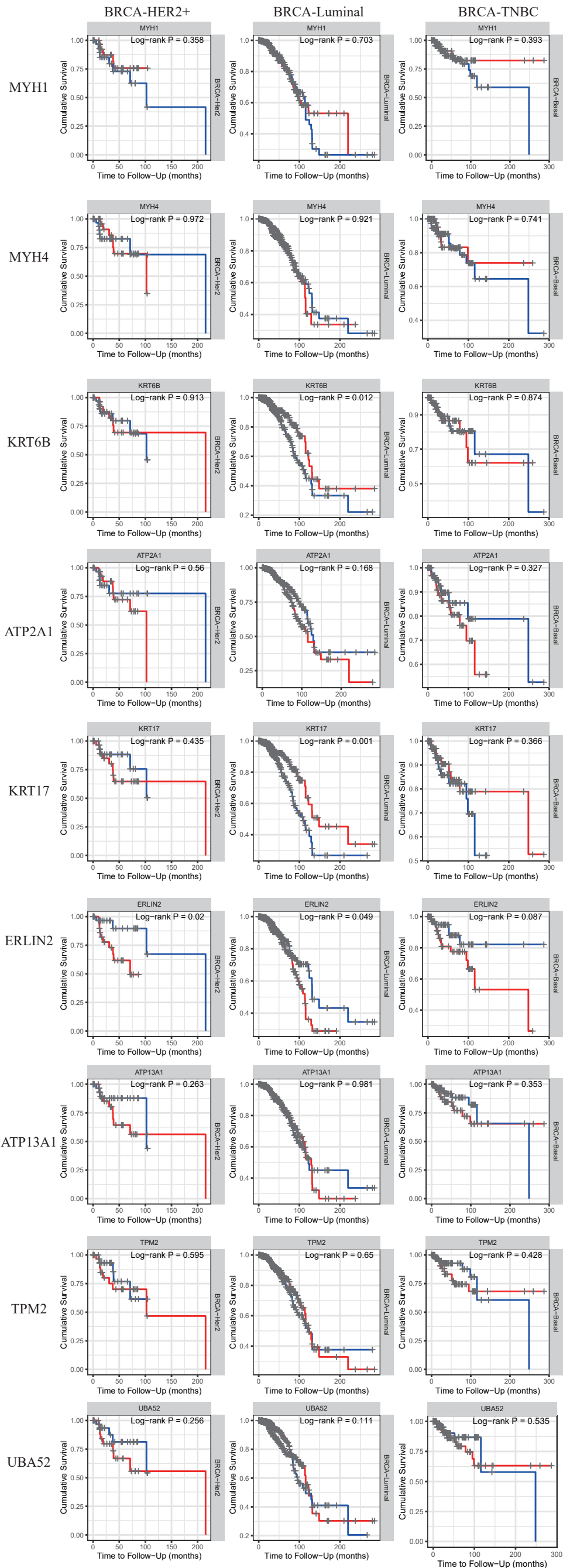

Supplement: Supplementary file 5 — supplementary Figure 4 [file 41420_2024_1804_MOESM5_ESM.pdf]

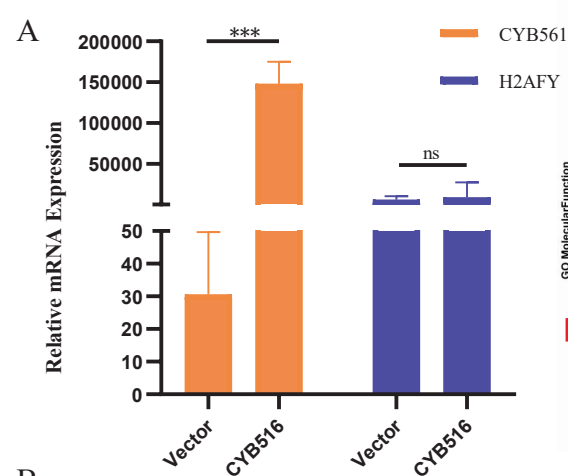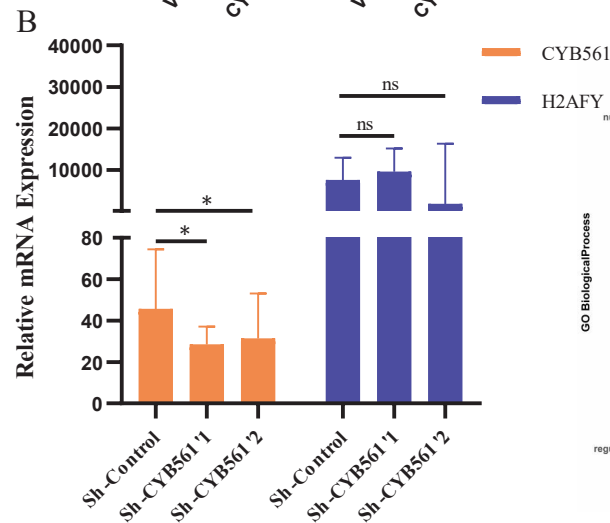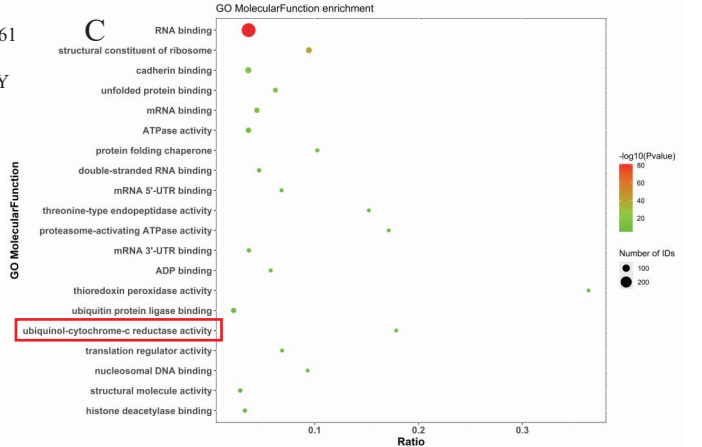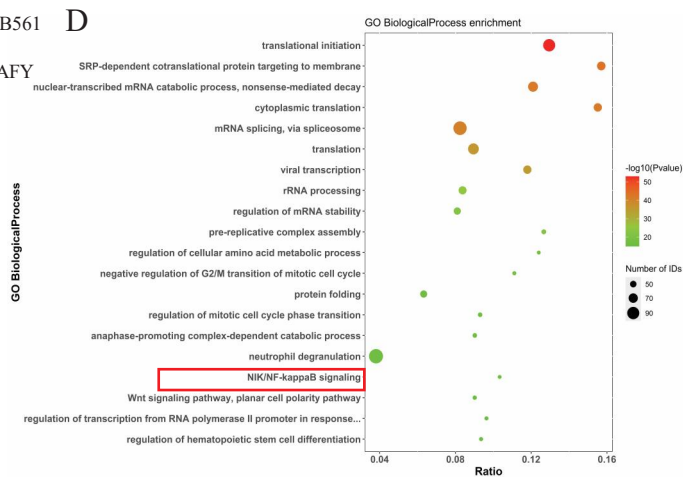

Supplement: Supplementary file 6 — supplementary Figure 5 [file 41420_2024_1804_MOESM6_ESM.pdf]
